# Network Adaptive Deadband: NCS Data Flow Control for Shared Networks

**DOI:** 10.3390/s121216591

**Published:** 2012-12-03

**Authors:** Miguel Díaz-Cacho, Emma Delgado, José A. G. Prieto, Joaquín López

**Affiliations:** 1 School of Computer Engineering, Computer Network Engineering, University of Vigo, 32004 Ourense, Spain; 2 School of Industrial Engineering, Control Engineering, University of Vigo, 36310 Vigo, Spain; E-Mails: emmad@uvigo.es (E.D.); jagprieto@uvigo.es (J.A.G.P.); 3 School of Industrial Engineering, Robotics, University of Vigo, 36310 Vigo, Spain; E-Mail: joaquin@uvigo.es

**Keywords:** deadband control, NCS, tele-operation, network protocols, sensors/actuators

## Abstract

This paper proposes a new middleware solution called Network Adaptive Deadband (NAD) for long time operation of Networked Control Systems (NCS) through the Internet or any shared network based on IP technology. The proposed middleware takes into account the network status and the NCS status, to improve the global system performance and to share more effectively the network by several NCS and sensor/actuator data flows. Relationship between network status and NCS status is solved with a TCP-friendly transport flow control protocol and the deadband concept, relating deadband value and transmission throughput. This creates a deadband-based flow control solution. Simulation and experiments in shared networks show that the implemented network adaptive deadband has better performance than an optimal constant deadband solution in the same circumstances.

## 1. Introduction

**Figure 1.**
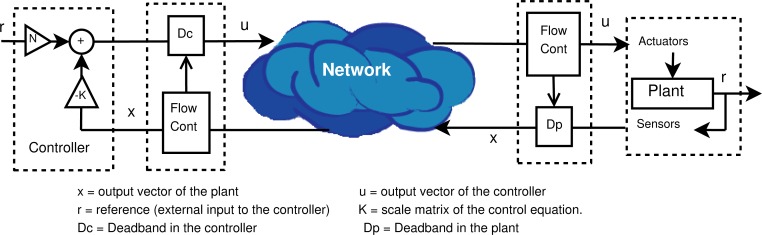
NAD basic scheme.

Control systems are basically composed by a controller and a plant, where the controller receives the information about the state of the plant, computes the new action and sends it to the plant. The controller uses the required hardware and software to obtain the control law. The plant uses actuators to apply the received actions from the controller, and uses sensors to measure the state of the plant (Figure 1). Sensors and actuators were traditionally connected to the control system through a dedicated network. If the control loop between controller and plant is closed through a shared digital communication network such as Internet, stability cannot be guaranteed because of variable communication delays and data packet loss. But the use of Internet as intermediate network has some advantages, such as the high availability, testability, simple installation as well as low cost. Networked Control Systems (NCS) cover tele-operation, tele-robotics and network-based control. The applications are in traffic control, robotics, domotics, tele-operation, in general Internet of things and can be extended to serve as remote back-up controllers for some critical plants in case of a disaster.

The adoption of TCP/IP protocol stack is mandatory to send packets across the Internet, and researchers should work with this restriction. Major contributions of the researches in NCS over Internet are in the controller-design, in designing new transport protocols (in layer 4 of the TCP/IP stack) or in the local segment of the extremes of the NCS. The Internet constraints for NCS (such as delay and packet losses) are basically caused by the queue size of the intermediate routers and the capacity of the network link between both extremes of the NCS. Variable delay and variable packet losses are mainly due to the unpredictable network occupation of the background traffic and the transmission rate of the NCS [1]. The transmission rate is closely related with the sampling period of the NCS and the network constraints [2]. A big effort is made to calculate the limits of the sampling periods that maintain the NCS stability, such as the *Maximum allowable sampling period* (or MASP) of an NCS (see the work of Nesić *et al*. [3] and references cited therein).

In our previous study [4], we conclude that an adequate transport scheme can solve the transmission rate problem in Internet-based NCSs. The transport scheme should have flow-control, best-effort and fairness features, as well as an NCS data priority system. Some transport protocols meet the first three specifications, but compete with control data scheduling strategies implemented in the control design, to solve the variable delay and packet losses problem.

In this work, a general scheme to combine control and network status is presented. The general scheme is implemented in a middleware solution that uses a transport protocol that satisfies the aforementioned features, in combination with the deadband concept that works as the NCS data priority system (see Figure 1 for a basic Linear Quadratic Regulator LQR control system). However, a more generic adaptive deadband solution may be implemented with other adaption law, mainly to optimize the power consumption, highly recommended in sensor networks.

The starting point to implement the proposed middleware is a properly designed NCS in the expected network scenario, taken into account delays and packet losses. Several works have been made to design such an NCS, using different strategies that will be described in Section 2.

The concept of deadband applied to NCS was initially studied by Otanez *et al*. [5]. NCS deadband focuses on the relative value of NCS data to be transmitted. In deadband sampling solution, a data is sent only if its difference with the last data sent is bigger than a defined value. This value is called *deadband*.

(1) $$DFi_{\mathrm{send}}=\left\{ \begin{array}{l} 1\;\text{if}\;\left\|x_{ls}-x\right\|\geq \delta \\ 0\;\text{if}\;\left\|x_{ls}-x\right\|<\delta \end{array}\right.$$

A deadband transmission filter can be formulated as in Equation (1), where *δ* is the deadband value, *x_ls_* is the last data sent and *x* is the candidate data to be sent. In Figure 1, *D_c_* and *D_p_* are the deadband filters for the controller and plant sides respectively.

Constant deadband solution attempts to minimize the throughput while maintaining acceptable performance for an NCS. However, a constant deadband that minimizes the throughput may not be suitable for networks with variable congestion scenarios, basically due to two remarks:

**Remark 1** *If the network is congested, a minimum transmission throughput can result in a reception throughput below the minimum required to maintain stability, due to packet loss.*
**Remark 2** *If the network is not congested, increasing the transmission rate improves the performance of the NCS, but the rate should stay below the available capacity on the network, because the delay increases due to saturation of the queues of the intermediate routers [4].*

The packet selection process that allows the deadband solution is suitable as a prioritization system for NCS data on shared networks, but does not take into account the state of the network to maximize the performance of NCS. This paper proposes a deadband modulation system taking into account the previous mentioned remarks. To module the deadband, a TCP-friendly flow control algorithm is used as transport protocol, and the complete middleware solution is called TCP-friendly NAD.

A flow is TCP-friendly if its arrival rate is approximately equal or slower than the rate of a conformant TCP connection under the same network conditions [6].

(2) $$\frac{1}{IPG}\equiv th{|}_{pkt/\sec}=\sqrt{3}/\left(RTT/\sqrt{2p}\right)$$

The conformant TCP throughput without retransmission follows Equation (2) for fixed packet size. In Equation (2), *p* represents the probability of packet losses and *RTT* the round trip time. *IPG* is an acronym for Inter-Packet Gap, which represents the time between packets sent, and *th* stands for throughput, in packets per second.

TCP-friendly requirements can be mandatory for new Internet protocols in order to share the Internet with good results. This feature can be exploited by other shared networks, and could be necessary for multi-agent systems.

**Figure 2.**
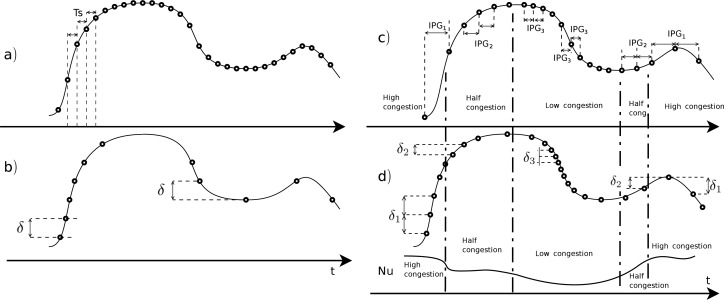
Points representing data transmission events for diverse transmission schedulers. (**a**) Fixed sampling rate with period *T_s_*; (**b**) constant deadband with value *δ*; (**c**) variable transmission rate with the Inter-Packet Gap (*IPG*) value depending on the transport flow control algorithm; (**d**) network adaptive deadband (NAD) tracking network usage *N_u_*.

The benefits of the proposed transport scheme can be seen in Figure 2. This figure shows a comparison between commonly used transmission schedulers and the NAD, where the points represent a transmission event.

- Figure 2(a) represents a transmission scheduler for a constant sampling rate with period *T_s_*, but without transport flow control or deadband.
- Figure 2(b) represents a transmission scheduler for a constant deadband sampling with a deadband value of *δ* according to Equation (1).
- Figure 2(c) represents a transmission scheduler based on a TCP-friendly transport protocol, with different network congestion scenarios, where *N_u_* represents the network use. In this figure, the *IPG* depends on the transport flow control algorithm, that generally depends on the network usage: with high congestion situations, the packet send period or *IPG* is high (*IPG*_1_), and it decreases in low congestion scenarios (*IPG*_1_ > *IPG*_2_ > *IPG*_3_).
- Figure 2(d) represents the proposed network adaptive deadband solution, a deadband scheduler based on the network usage *N_u_*. In this subfigure the value of the deadband depends on *N_u_*. It will have a larger deadband (*δ*_1_) in a high congestion network environment, and a smaller deadband (*δ*_3_) in a low congestion situation. Note that *δ*_1_ > *δ*_2_ > *δ*_3_.

The main reason for using this deadband solution rather than the constant deadband, amplitude relative deadband [7] or pure transport protocol is that the reduction of data sent caused by the constant deadband is not always required on shared networks (see remark 2). Besides, this reduction is at the expense of an increment in the NCS error or a decrement in the psychophysical perception of the tele-operated system. At the same time, packet loss in the case of constant deadband can cause instabilities if the transmission scheduling rule is in the low limit of acceptable throughput (see remark 1). A pure transport protocol such as the trinomial algorithm, adapted to the network scenario, can share the network with fairness, but it does not guarantee any of the NCS requirements to maintain stability.

On the other hand, a schema that combines the two aforementioned solutions can share the network with fairness requirements, and at same time, they can maintain the best available performance in a variable network scenario.

The paper is organized as follows. Motivation, originality and introduction to preliminary concepts such as TCP-friendly and deadband are advanced in this section. An overview of the related work is given in Section 2. Section 3 shows a general scope of Network Adaptive Deadband and defines the different elements involved in the scheme. The proposed TCP-friendly based deadband control is presented in Section 4 and its application to a tele-operated gantry-crane is showed in Section 5. Simulation and experimental results that compare constant deadband and adaptive deadband in a shared network are presented in Sections 6 and 7 respectively. Finally, the conclusions and future work are described in Section 8.

## 2. Related Work

Network and control system relationship in an Internet based NCS or in tele-operation systems is usually limited to adapt the controller design to several network variables (such as the delay constraint, the delay variation and the number of consecutive losses).The common solutions for the control system use the network as a passive element, and in the case of Internet, solve the transport problem using the UDP transport protocol. On the other hand, common network solutions for NCS investigate the creation of new protocols and data flow control algorithms. In this case, control systems are used as applications without considering the contents of the data to be transmitted. Modern techniques have emerged with the generic name of co-design that try to model and integrate the network in the control system. Some authors refer generically to this topic as Network Based Control (NBC). There are different solutions to this problem that we classify in the following four approaches.

### 2.1. Packet and Deadband Based Control

This line scans the contents and structure of *NCS data packets* to optimize the network performance and efficiency [8,9]. These methods allow to work on predictive control, because the controller (or the plant) can send actual, past and future *NCS data*, in the same packet. Thus, if the data is lost or not sent, the other extreme has predictive information received in previous packets [1,10].

Deadband control applied to NCS focuses initially on modeling Networked Control Systems [5] analyzing the performance effects of limiting packet transmission to reduce the network traffic. This makes the communication medium more efficient, and time delays are minimized.

In [7] a passivity approach for deadband control is made for haptic tele-operation, and later, perception Weber-inspired deadband approaches were made [11]. In [2] a study about the relationship between the sampling rates of a control system, and the transmission rates of a local communication network, is made. A similar solution to the one proposed in this paper has been presented in [12]. However, the work in [12] was made specifically for wireless sensor networks; in [13], the predictive deadband allows to study the influence of different factors, such as the CPU load, on the efficiency of deadband sampling. These works focus the deadband study to improve the performance of sensor networks, optimizing the network and/or the power consumption of the whole system.

### 2.2. Codesign of Control and Transmission Rate

This line of work studies protocols, middleware systems and control theory. The work of Nesic and Teel [3,14] focuses on the transmission rate (or sampling) problem of nonlinear systems.

In event-triggered control, the control input is updated when a certain condition of the state of the plant is met. For example the work of P. Taboada and M. Mazo in [15,16] and the references therein present very interesting results in modeling sampling rate (or transmission rate) and control system. In self-triggering control [17], the controller calculates the next sampling-time as a function of the state of the plant. The target of the triggering control is to minimize the energy requirements, without compromise the performance of the control system.

### 2.3. Codesign of Control and Gain

In [18] a middleware system that modifies control gains in PID controllers is studied. The system takes into consideration the network status. The controller output algorithm depends on the remote plant and the controller global configuration. The state of the network is obtained by sending probe packets. More recently, in [19] a network delay-dependent gain scheduling law is used on an Ethernet shared network.

### 2.4. NCS Specific Transport Protocols

In the transport layer, there are some solutions related to the UDP protocol, developing flow-control and TCP-friendly features, such as TFRC [20], DCCP [21] or the trinomial algorithm [22]; Recently, Wirz *et al*. have worked in transport protocols for robotics, such as the Bidirectional Transport Protocol (BTP) [23]. BTP is an end-to-end flow control protocol that minimizes the round trip time while maximizing the transmission rate. The Tele-operation Data Transport Protocol (TDTP) [9] focuses on the bidirectionality of the NCS data flow to improve the efficiency of the transmission rate. Bidirectionality of the NCS systems in transport protocols is used to determine the network status in the packet header of the reverse flow.

In the Link Layer of the OSI model, CAN, HART or EtherCat, and the technologies like Ethernet, Wifi 802.11, bluetooth or ZigBee are suitable as the local segment of the Internet NCS connection with an appropriate gateway to the Internet link.

To our knowledge, no work has been done to relate deadband sampling and transport flow control algorithms.

## 3. Network Adaptive Deadband Proposed Scheme

**Figure 3.**
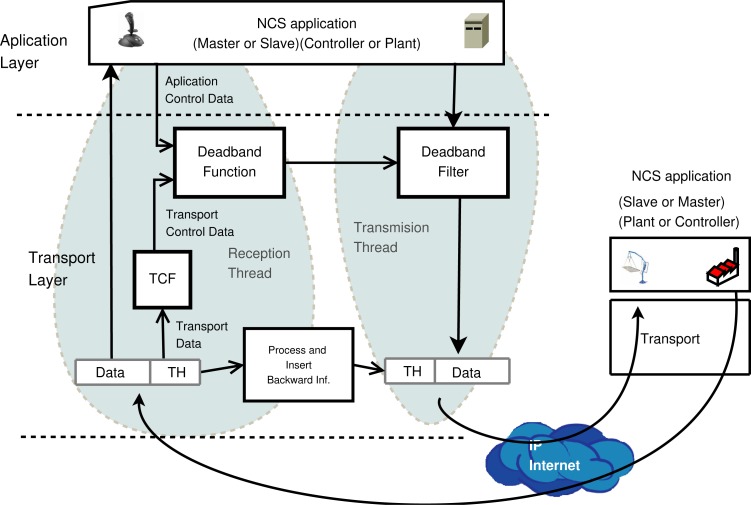
Network Adaptive Deadband general middleware.

The solution presented here is a middleware that combines transport and application layer functionality. The application layer will deliver NCS status data to the transport layer and the transport layer looks to the application data fields. Figure 3 shows a detailed box diagram of the functionality and data flow between the layers. Network status is calculated using a bidirectional transport architecture, where the network information is inserted in the transport header.

The middleware uses two threads. The *Reception thread* delivers application data directly to the NCS. The *Transport Data* (TD) of the *Transport Header* (TH) are processed in the transport layer by the *Transport Control Function* (TCF), where the flow control algorithm is implemented. The TCF generates *Transport Control Data* (TCD) that includes processed information regarding the network status. This TCD is provided to the *Deadband Function* (DF), which relates throughput and deadband. Optionally, application control information such as error, *Average Error* (AE) or predictive data, can be used as DF input. These data are called *Application Control Data* (ACD).

The *Transmission thread* filters NCS data with the *Deadband Filter* (DFi), which uses the deadband value obtained dynamically by the DF in the reception thread. Deadband filter is implemented using the sending event function defined in Equation (1). The proposed adaptive deadband formulates the deadband function as in Equation (3), where *th*, *RTT*, *p* are the throughput, round-trip time and packet losses respectively ; these variables are used as TCD data. Average error (*AE*) or instantaneous error (*error*) are proposed ACD data.

(3) $$\begin{array}{l} \delta =f\left(TCD,ACD\right)\\ \delta =f\left(\left(th,RTT,p\right),\left(AE,\mathrm{error}\right)\right) \end{array}$$

## 4. TCP-Friendly Adaptive Deadband

In this section, the transport control scheme and the deadband function for the TCP-friendly Adaptive Deadband are described.

### 4.1. Transport Control Scheme

The implemented transport scheme focuses on the flow control algorithm and on the use of bidirectionality in NCS data flows.

The transport control function (*TCF*) implemented is the trinomial flow control algorithm [22]. The trinomial algorithm calculates the *IPG* and differentiates increment and decrement transmission rate intervals. To compute the transmission rate, the algorithm uses three parameters (*α*, *β*, *γ*), and the RTT. The RTT is measured using timestamps. The sender selects an increment or decrement interval depending on a backward packet loss tag. This tag, called Backward Congestion Notification (BCN), is inserted in the reverse flow by the receiver and received by the sender. The details of this tag and the way it is included in a bidirectional data flow, are explained in our previous work [9,24].

**Figure 4.**
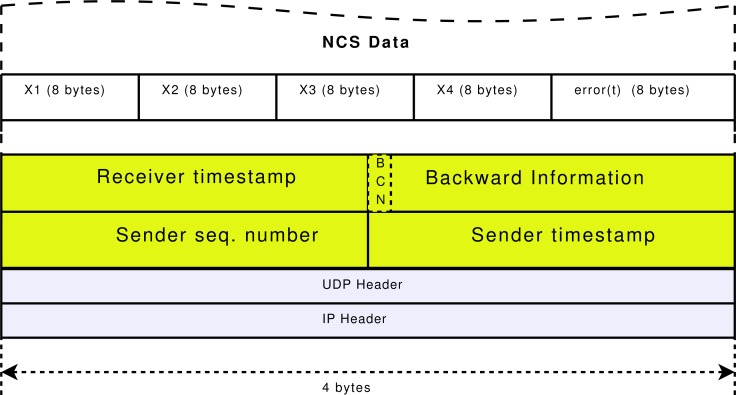
Bidirectional transport header for trinomial algorithm and data structure for tele-operated gantry crane.

In the direct flow between sender and receiver, the receiver detects the loss of packets by computing a sequence number and reports it to the sender setting the BCN tag to one (*BCN* = 1). If there are not packet losses, the BCN tag is set to zero (*BCN* = 0). Figure 4 shows the transport header format of the implemented trinomial algorithm, with the BCN tag, the sender sequence number and sender and receiver timestamps.

(4) $${\mathrm{IPG}}_{i+1}=\{\begin{array}{c} \frac{{\mathrm{IPG}}_{i}\cdot W}{{\mathrm{IPG}}_{i}+W}\text{if}\;BCN=1\\ \frac{{\mathrm{IPG}}_{i}}{\beta }\text{if}\;BCN=0 \end{array}$$

Equation (4) shows the way the transmission rate is decremented (with *BCN* = 1) or incremented (with *BCN* = 0), where *W* > 0 is a variable depending on *γ*, *α* and *RTT*. Parameter *β* < 1 is an initial parameter. To know how this algorithm is determined and the details of how it works, see the work of Liu *et al*. [22].

### 4.2. Deadband Function

In this work, we have chosen as deadband function the relationship between transmission rate (or throughput *th*) and deadband (*δ* = *f*(*th*) ≡ *δ_th_*). The implementation of the trinomial algorithm presented in Section 4.1 meets the TCP-friendly requirements, and gives a way to calculate the throughput *th* by calculating the inter packet gap *IPG* = 1/*th*.

**Figure 5.**
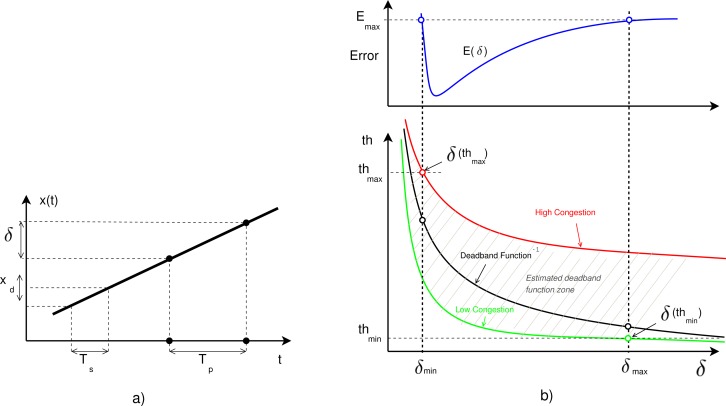
(**a**) Linear approach of *th*(*δ*); (**b**) Throughput and error versus deadband.

Obtaining an analytical solution to the relationship between *th* and deadband (*δ* = *f*(*th*) ≡ *δ_th_*) is complex and can be attempted using rate-distortion theory [25] or a simple linear approach. From the rate-distortion theory, the throughput-deadband relationship is a continuous, monotonically decreasing convex function, and tends to have very similar forms in real implementations. Figure 5(b) shows these relationships. Experiments, simulations and related literature [1,2,5,7,25–27] show that this relationship always has that form.

The purpose of this work is not to determine an exact representation of *δ_th_* but a deadband function easy to implement, maintaining the lower and upper bound of the transmission rate to achieve the performance of the control system. A similar simulated method for Wireless Sensor Networks, was used in the work of Xia *et al*. [12]. Lower and upper bounds of the throughput can be obtained by diverse methods or analysis, such as the ones presented by Tatikonda *et al*. [28]. From [4], in Internet with TCP congestion, the upper limit of the transmission rate should be smaller than without congestion (*th_max_*); with UDP congestion, the lower limit should be bigger than without congestion (*th_min_*). However, the DF has the form in Figure 5(b), where the bounds are also represented.

The linear approach of the DF can be obtained with a basic analysis for a linear and uniform output signal (*x*(*t*)) in one extreme of the NCS with high sampling rate (*sr*), as seen in Figure 5(a), where *T_p_* ≡ *IPG* is the transmission period, *T_s_* ≡ 1/*sr* is the sampling period, *δ* is the fixed deadband value and *x_d_* is the difference between consecutive sampled data. In this case, these parameters satisfy Equation (5).

(5) $$\frac{1}{T_{p}}=\mathrm{th}=\frac{x_{d}}{\delta }\cdot \frac{1}{T_{s}}$$

However, in packet based NCS, the output is a discrete function. Therefore, in order to satisfy $\underset{\delta \to 0}{\text{lim}}th=sr$, the previous equation has to be changed to:

(6) $$\mathrm{th}=\frac{x_{d}}{\delta +x_{d}}\cdot sr$$

In dynamic environments, the output signals are nonlinear and discontinuous, and the DF *δ_th_* changes dynamically with the environmental states. To reflect this non-linearity and discontinuities in output signals, the transmission rate is estimated as an average of the different linear functions adding a new parameter *n*_1_ in Equation (6). The transmission rate (*th*(*δ*)) is then characterized as a function of the system and controller dynamics, reference trajectory, and the chosen deadband [2] in a simple way.

(7) $$\mathrm{th}\left(\delta \right)=\frac{c}{\delta^{n_{1}}+c}\cdot sr$$

In Equation (7), the parameter *c* is directly related with the average of the modulus of the output signal variation (*x_d_*), and should always have a positive value. The parameter *n*_1_ is obtained by simulation and is in the range of 0 < *n*_1_ < 1.

The function *δ_th_* should be have a maximum deadband *δ_max_* and a minimum deadband *δ_min_* to maintain acceptable performance. The maximum deadband depends on the minimum acceptable throughput *th_min_* = 1/*IPG_max_* and a measure of maximum allowed error of the NCS (*E_max_*), as seen in Equation (8).

(8) $$\delta_{\max}=\min\left\{\delta_{{th}_{\min}},\delta_{E_{\max}}\right\}$$

The minimum deadband depends on the maximum acceptable throughput *th_max_*, depending on the queue size of the intermediate routers of the network [4].

These values are important to limit the range of the deadband function and to guarantee the stability of the NCS.

Figure 5(b) represents the typical relationship between deadband and throughput (*th*(*δ*)) or error (*E*(*δ*)), where deadband limits are also reflected. The *th*(*δ*) function varies with the traffic load of the shared network, as seen in the figure. The error *vs*. deadband curve is represented as *E*(*δ*) and his formulation is out of the scope of this paper. Equation (7) corresponds to the *DF*^−1^ throughput curve of the Figure 5(b).

For fixed packet size, *th* of Equation (7) can be replaced by 1/*IPG*, and the deadband function DF can be represented as:

(9) $$DF=\delta_{IPG}=\left\{ \begin{array}{l} \delta_{\min}\;\;\text{if}\;\delta \leq \delta_{\min}\\ \delta ={\left[c\left(IPG\cdot sr-1\right)\right]}^{1/n1}\;\text{if}\;\delta <\delta_{\max}\\ \delta_{\max}\;\;\text{if}\;\;\delta \geq \delta_{\max} \end{array}\right.$$

**Figure 6.**
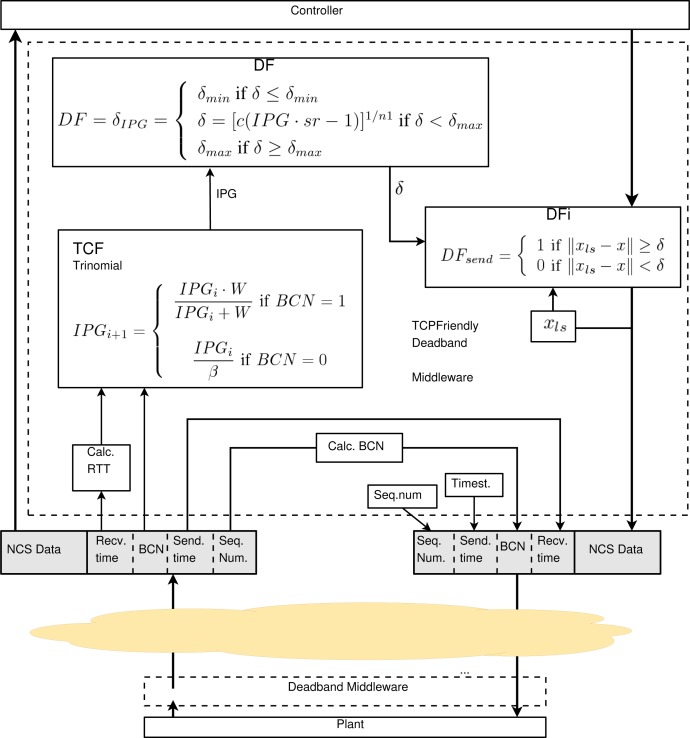
TCP-friendly adaptive deadband.

Figure 6 represents the TCP-friendly Adaptive Deadband scheme on the controller side, but it is similar on the plant side.

#### 4.2.1. Determining the Optimal Constant Deadband

These approaches can be used to determine the constant deadband filter, by using the procedure presented in [5], and summarized below:

(10) $$\begin{array}{l} P\left(\delta \right)=K_{1}\cdot th_{n}\left(\delta \right)+K_{2}\cdot E_{n}\left(\delta \right)\\ P\left(\delta_{c}\right)=\min_{\delta }\left\{K_{1}\cdot th_{n}\left(\delta \right)+K_{2}\cdot E_{n}\left(\delta \right)\right\} \end{array}$$

Constant deadband selection should follow the criteria in Equation (10), where *P*(*δ*) is a deadband dependent global performance function and *δ_c_* is the optimal deadband value. Throughput and error should be normalized (*th_n_* = *th/sr* and *E_n_* = *E/E_max_*). *K*_1_ and *K*_2_ are weighting and scale factors.

The results obtained in simulation show that optimal deadband for an NCS varies with network conditions and reference signals.

#### 4.2.2. Stability Issues

There are some stability analyses for NCS using deadband. In [5] the deadband is modeled as a vanishing perturbation. Then, if the original system is exponentially stable, the existence of a Lyapunov function is guaranteed in the perturbed system. Using the BIBO (Bounded Input Bounded Output) concept for NCS with LQR controllers, we can use the principle that any stable and linear system with bounded inputs generates bounded outputs. In a similar way, Lian *et al*. in [2] demonstrate stability for networked multi-agent systems that use deadband in the communication modules. For adaptive but bounded deadband, a similar principle can be used.

For tele-operated systems, the passivity approach based on scattering transformation [7] may be used to demonstrate stability using a bounded deadband.

## 5. TCP-Friendly Adaptive Deadband for a Tele-Operated Gantry Crane

The proposed schema has been tested in some control systems in our laboratory, such as a Mass-Spring and a gantry crane with a PID controller.

To present a practical implementation of the proposed solution, we use an application where the tele-operated slave is a laboratory crane, which is a scale model of a typical industrial gantry crane, and the master is a human tele-operator. The crane is equipped with actuated motors and sensors. Although the true dynamics of the crane are nonlinear and coupled, it will be assumed that the crane movements are smooth and slow, and that the swing angles of the load are small enough. Under these assumptions, the linearization of the crane is adequate to model the system. Furthermore, in this situation, the *x*, *y*, *z* dynamics become uncoupled. The vertical (or *z*) dynamic is controlled by the motor that lifts the rope. The *x* and *y* dynamics are formally equivalent and contain the load balance or swing. We will concentrate our attention on the *x* (or *y*) movement of a cart, provided by a motor, hanging a swinging load, not actuated, with a rope of fixed length.

The relation between the cart position *x_s_*(*t*), the swing angle *θ_s_*(*t*) and the force *F_s_*(*t*) exerted by the cart motor is given by:

(11) $$\begin{array}{l} F_{s}\left(s\right)=M_{s}\left\lfloor \left(s^{2}+B_{g}s\right)\cdot x_{s}\left(s\right)-M_{\mu }\cdot g\cdot \theta_{s}\left(s\right)\right\rfloor \\ F_{s}\left(s\right)=\left(-L_{0}M_{s}\right)\left(s^{2}+B_{0}s+\omega_{0}^{2}\right)\cdot \theta_{s}\left(s\right) \end{array}$$

where *M_s_* is the mass of the cart or trolley, $\omega_{0}=\sqrt{\left(1+M_{\mu }\right)\frac{g}{L_{0}}}$, with *L*_0_ the rope length, $M_{\mu }=\frac{M_{\mathrm{load}}}{M_{s}}$ with *M_load_* the load mass and *B_g_*, *B*_0_ the damping coefficients. We have used the subscript *s* to denote that this is the slave system in the tele-operation scheme. A state-spaced model can be defined considering the state vector *z*(*t*) ∈ ℝ*^n^* as:

(12) $$z={\left(\begin{array}{cccc} z_{s}\left(t\right) & v_{s}\left(t\right) & \theta_{s}\left(t\right) & \omega_{s}\left(t\right) \end{array}\right)}^{T}$$

where *x_s_*, *v_s_*, *θ_s_* and *ω_s_* are the slave position, velocity, swing angle and angular velocity. In state-space the system is described with the following expressions:

(13) $$\begin{array}{l} \left(\begin{array}{c} {\dot{z}}_{1}\\ {\dot{z}}_{2}\\ {\dot{z}}_{3}\\ {\dot{z}}_{4} \end{array}\right)=\left(\begin{array}{cccc} 0 & 1 & 0 & 0\\ 0 & -B_{s} & M_{\mu }\cdot g & 0\\ 0 & 0 & 0 & 1\\ 0 & 0 & -\omega_{0}^{2} & -b_{0} \end{array}\right)\cdot \left(\begin{array}{c} z_{1}\\ z_{2}\\ z_{3}\\ z_{4} \end{array}\right)+\left(\begin{array}{c} 0\\ \frac{1}{M}\\ 0\\ -\frac{1}{L_{0}M_{s}} \end{array}\right)F_{s}\left(t\right)\\ \left(\begin{array}{c} y_{1}\\ y_{2} \end{array}\right)=\left(\begin{array}{cccc} 1 & 0 & 0 & 0\\ 0 & 0 & 1 & 0 \end{array}\right)\left(\begin{array}{c} z_{1}\\ z_{2}\\ z_{3}\\ z_{4} \end{array}\right) \end{array}$$

Or in compact form:

(14) $$\begin{array}{l} \dot{z}\left(t\right)=Az\left(t\right)+BF_{s}\left(t\right)\\ y\left(t\right)=Cz\left(t\right) \end{array}$$

For the implemented dynamic model of the 3D crane Inteco Ltd. the numerical values are:

(15) $$\dot{z}\left(t\right)=\left(\begin{array}{cccc} 0 & 1 & 0 & 0\\ 0 & -18.3 & 0.959 & 0\\ 0 & 0 & 0 & 1\\ 0 & 0 & -9.5 & -0.003 \end{array}\right)z\left(t\right)+\left(\begin{array}{c} 0\\ 7.2\\ 0\\ -0.95 \end{array}\right)\cdot F_{s}\left(t\right)$$

The controller at the master side receives the state information from the slave side and implements a linear quadratic regulator LQR for a steady-state optimal control. The selection of weighting matrices *Q*_1_ and *Q*_2_ is made so that the cost function is:

(16) $$\begin{array}{l} J=\int_{0}^{\infty }\left(z^{T}Q_{1}z+F_{s}^{T}Q_{2}F_{s}\right)dt\\ Q_{1}=\left(\begin{array}{cccc} 1 & 0 & 0 & 0\\ 0 & 0 & 0 & 0\\ 0 & 0 & 25 & 0\\ 0 & 0 & 0 & 0 \end{array}\right),\;\;\;Q_{2}=2.0408 \end{array}$$

Moreover we introduce the Pincer procedure, a modification to the performance criterion that allows a degree of direct control over the settling time:

(17) $$\begin{array}{l} J_{\alpha }=\int_{0}^{\infty }\left(z^{T}Q_{1}z+u^{T}Q_{2}F_{s}\right)e^{2\alpha t}dt=\int_{0}^{\infty }\left(w^{T}Q_{1}w+u^{T}Q_{2}\right)dt\\ w\left(t\right)=e^{\alpha t}z\left(t\right),\;\;\;u\left(t\right)=e^{\alpha t}F_{s}\left(t\right) \end{array}$$

From the definitions in Equation (17) the revised system for use in a LQR computation for the feedback gain matrix is:

(18) $$\begin{array}{l} \dot{w}\left(t\right)=\left(\alpha I_{4x4}+A\right)w\left(t\right)+Bu\left(t\right)\\ u\left(t\right)=N\cdot r\left(t\right)-K\cdot w\left(t\right) \end{array}$$

where *r*(*t*) is the reference for the controller. If the designer selects the settling time *t_s_* = 3, within which all states should settle to less than 2%, it is obtained:

(19) $$\begin{array}{l} z\left(t\right)=z\left(0\right)e^{-\alpha t}\\ z\left(t_{s}\right)=z\left(0\right)e^{-\alpha t_{s}}\leq 0.02\cdot z\left(0\right)\Rightarrow \alpha >\frac{L_{n}50}{t_{s}} \end{array}$$

The slave and the LQR controller by the master are using now the equations in Equation (11). After some trials, the requirements of the system are accomplished with a control law given by *K* = (12.1661, 0.6646, −22.8596, −3.4107) and *N* = 12.1661.

**Figure 7.**
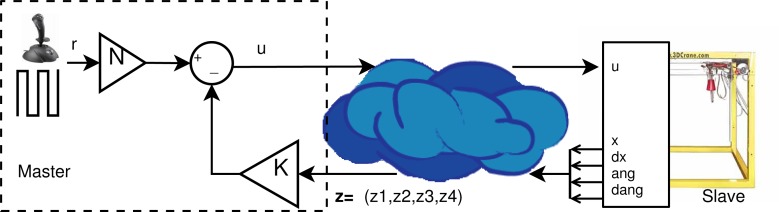
Tele-operated gantry crane.

Figure 7 shows a representation of the tele-operated gantry crane, simulated in this work.

### 5.1. Adaptive Deadband Function

The deadband function should be determined for the master and the slave sides, but as illustrative example, only the process for the master side is presented. In this process, the slave side remains with a constant deadband of value 0.

**Figure 8.**
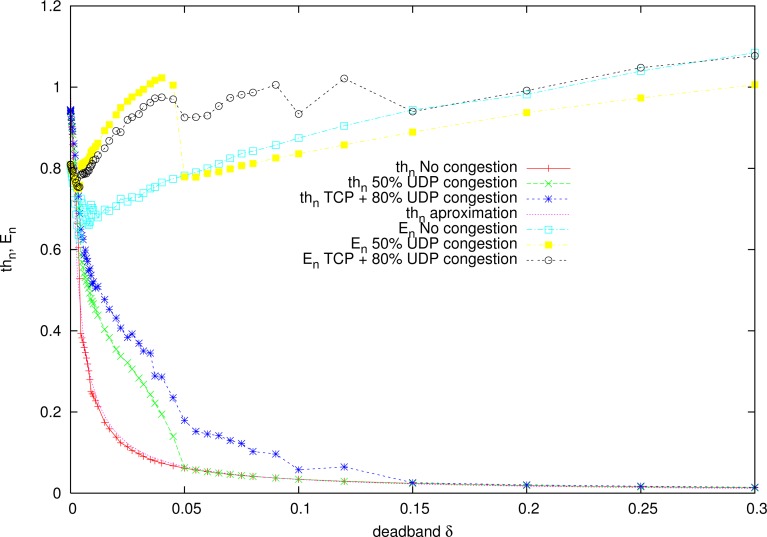
Throughput (*th_n_*) and average error (*E_n_*) response *vs*. deadband (*δ*) with different network status for a tele-operated gantry crane. Reference signal is $10\sin\left(2\pi \frac{1}{15}t\right)$.

Figure 8 represents the experimental functions *δ_th_* and *δ_E_* for the network conditions of the experiments, using the gantry crane with sinusoid reference signal of amplitude 10 and period 15 s. The approximate functions given in Equation (7) are also represented.

The function *th*(*δ*) parameters are approximated as *c* = 0.0035 and *n*_1_ = 1. For higher reference periods, the deadband function parameter *c* should be decreased, and parameter *n*_1_ varies with more abrupt signals, such as a square signal.

The normalized function is:

(20) $${th}_{n}\left(\delta \right)=\frac{0.0035}{0.0035+\delta }$$

Therefore, in Figure 8 for *E_max_* = 2.5 (*E_n_* = 1), the deadband has an approximate value of *δ_max_* = 0.25, and *δ_min_* = 0, for the maximum throughput *th_max_* that equals the sampling rate of 1, 000 pkt/s.

The deadband function DF for the studied tele-operated gantry crane is:

(21) $$\delta_{IPG}=\left\{ \begin{array}{l} \delta =0.0035\cdot \left(IPG\cdot 1000-1\right)\text{if}\;\delta <0.25\\ 0.25\;\text{if}\;\delta >0.25 \end{array}\right.$$

On the slave side, using the same approximation methodology, the parameters *c* and *n*_1_ are the same than on the master side, but the function *E*(*δ*) remains approximately constant. Therefore, a value of *δ_max_* = 10 can be adopted.

### 5.2. Constant Deadband for a Gantry Crane

**Figure 9.**
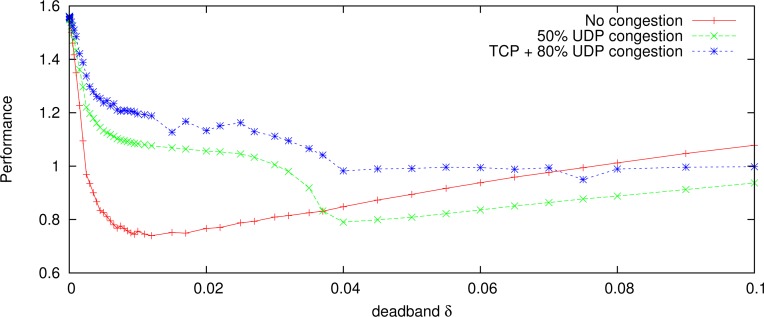
Constant deadband, experimental and approximated for a gantry-crane. Reference signal is $10\sin\left(2\pi \frac{1}{15}t\right)$.

The same reference signals are used to obtain the constant deadband. Results obtained in simulation and approximated minimum are shown in Figure 9 for some network congestion situations. The minimum is within the range of 0.01 < *δ_c_* < 0.05. In the Figure 9, the optimal constant deadband value is approximately *δ_c_* = 0.02145 for a sinusoid reference signal with period 15 s.

## 6. Simulated Example: Sharing the Network

The simulations in this section compare constant deadband (CD) with network adaptive deadband (NAD) in different network scenarios. The reason to make this comparison is to show the effectiveness of the NAD solution with respect to the CD solution in scenarios with high congestion when more than one NCS is used.

Simulations were made with two tele-operated gantry cranes. The two systems use different reference signals. The gantry crane 1 (gc1) uses a sinusoid reference signal with period 15 s and amplitude 10, and gantry crane 2 (gc2) uses a triangle reference signal with period 18 s and amplitude 10. Both reference signals are continuous and slow and have similar deadband parameters.

**Figure 10.**
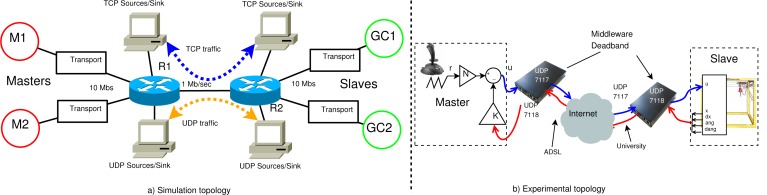
Simulation (**a**) and Experimental (**b**) topologies.

We use the co-design framework in [4] based on the simulation suite NS2. The network topology implemented is represented in Figure 10. The transport boxes in the figure can use a configured NAD transport schema, a constant deadband transport schema or a pure bidirectional transport protocol.

To simulate the Internet, we use a scenario with TCP and UDP traffic from diverse sources. The network topology uses a bottleneck trunk link between two routers with 1 Mbps capacity. The access links capacity to the routers is 10 Mbps. All links are symmetric. The two routers implement a Drop-Tail queue with a queue limit of 15 packets. Simulations were performed over a total of 100 s with hard bottleneck congestion of 80% due to UDP and TCP traffic, but no UDP congestion is simulated between 20 s and 40 s in both directions to show Best Effort characteristics.

A comparison between the optimal constant deadband functions and the adaptive deadband functions is made, with deadband function parameters deduced in Section 5, as shown in Table 1.

**Figure 11.**
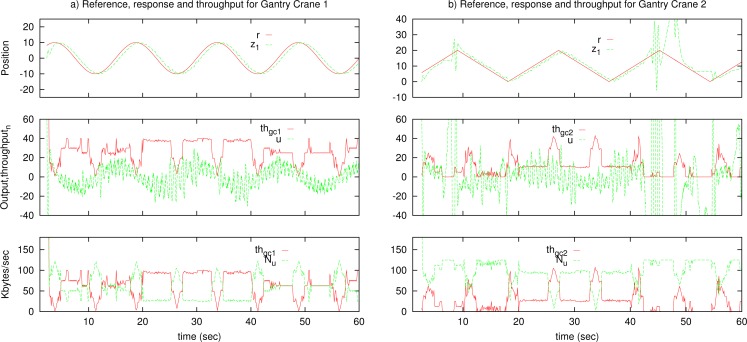
Simulation results for constant deadband.

**Figure 12.**
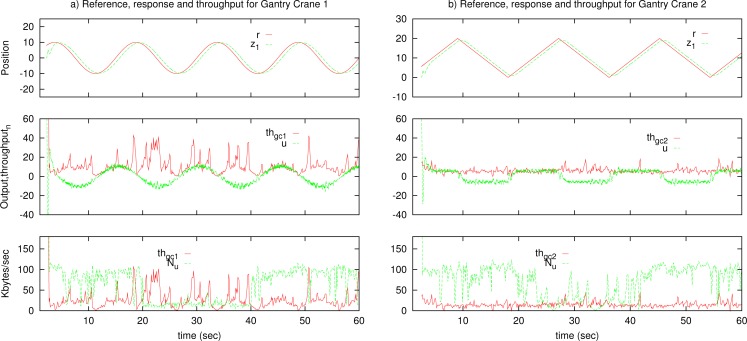
Simulation results for Network Adaptive Deadband.

The results are presented in Figure 11 for constant deadband and in Figure 12 for adaptive deadband. In the figures, the top graphs represent reference position of the master (*r*), and output of the slave (*x*_1_). The middle graphs represent the output of the master (*u*) compared with a suitable scaled throughput from the master to the slave, for clarity purposes. The bottom graphs represent the throughput of the tele-operated system from the master to the slave (*th_ms_*), compared with the remaining use of the network *N_u_*. Figure 11 in the constant deadband case shows that the two data flows compete to gain the network resources, and one of them (or the two) can be unstable. Using the sinusoid reference signal, the throughput is low on the top and bottom parts of the signal because of slow variation, and the gantry crane 2 has problems to maintain stability.

In Figure 12, with high variations of the master output, the bandwidth consumption of the system is high, until the output stabilizes, but remains fair with the rest of network traffic.

**Figure 13.**
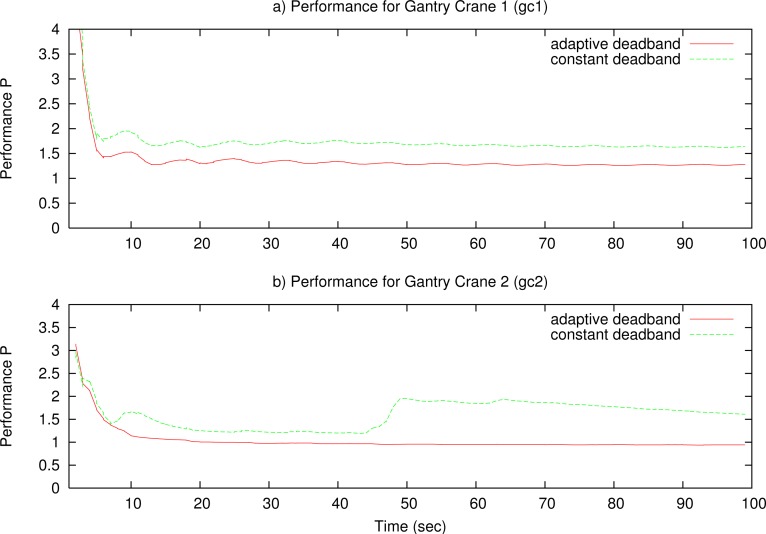
Performance comparison between optimal constant deadband and network adaptive deadband in the simulation.

Finally, Figure 13 shows a performance (Equation (10) with *K*_1_ = *K*_2_ = 1) comparison between TCP-friendly adaptive deadband and optimal constant deadband for the two gantry cranes. The results show that the adaptive deadband has better performance than the constant deadband for the long time operation.

## 7. Experimental Results

To validate the effectiveness of the proposed solution, NAD middleware was implemented in a real Internet scenario between an emulated Master through a normal ADSL Internet access and an emulated Slave through a high capacity Internet access in a University laboratory. The transport protocol used is presented in [9], based on a bidirectional implementation of the trinomial algorithm. The transport packet header is over the UDP protocol as shown in Figure 4, where the data structure is also depicted.

Experimental platform is presented in Figure 10(b), where the plant and controller are the emulated gantry crane and the LQR controller described in Section 5. The emulated system uses a triangle reference signal with period 18 s and amplitude 10. Similar transport parameters as gc2 in the simulated scenario of Section 6 were used. The parameters are represented in Table 1. Measured network conditions between both extremes have an average RTT of 43*msec* and 20% packet losses at a transmission rate of 200*pkts/s* with 100*bytes* packet size.

**Figure 14.**
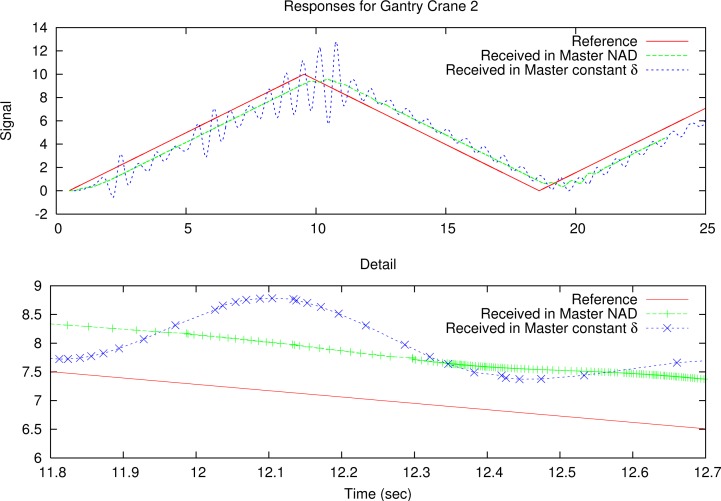
Compared results between NAD and constant deadband in the real Internet link for the gantry crane and the LQR controller.

The experiment compares the same tele-operation system with different transport solutions, in particular with an only TCP-friendly transport protocol, with the constant deadband solution and our NAD solution. Results are not presented graphically for the TCP-friendly transport protocol because the tele-operated system becomes unstable. Graphical results for the other cases are presented in Figure 14.

The results show that the NAD has better performance than constant deadband. In some circumstances, with high congestion, constant deadband can become unstable while NAD maintains stability.

## 8. Conclusions and Future Works

This paper proposes a new middleware solution to improve long-time operation of an NCS through the Internet or any shared network based on IP technology. This solution is useful for sensor networks where the network use or the power consumption is of special interest. The proposed middleware takes into account the network status and the NCS status to decide whether to send the output of the system. In that way, the network can be shared more effectively between several NCS or other bidirectional real-time flows, and the middleware can be used as a complement to a well-designed NCS on an unpredictable shared network. In this case, simulation and experimental results show that the proposed solution has better performance than a TCP-friendly transport protocol or a constant deadband solution.

The proposed scheme, called Network Adaptive Deadband or NAD, is a solution for network and control integration, using a simple relationship between deadband and TCP-friendly transport throughput. An experimental procedure is presented to estimate this relationship. Simulation and experimental results show that the middleware presents good performance in any network conditions, with a TCP-friendly transport control protocol applied to an estimated deadband function.

The results are of interest to maintain NCS stability and TCP-friendly flows, through IP-based networks. TCP-friendly protocols and deadband sampling do not ensure by itself best network and control system performance, but together they may give enough reasons to improve the use of the network and to maintain the control system stability. The utility of the network adaptive deadband is not restricted only to IP network applications, but in fact is also applicable to collaborative robotics in local networks, sensor networks, MMMS tele-operation or plants with several data flows.

The simulation and experimental results show that the middleware presents good performance in any network conditions, with a TCP-friendly transport control protocol applied to an estimated deadband function. The results evidence that network adaptive deadband is a good solution to share a public network as Internet for several NCS. The optimal constant deadband criterion can be adequate when there are no other NCS data flows that can collapse the network, but can be inefficient and generate instability caused by non-regulated flow control. Adaptive deadband can regulate the data flow depending on network status, and permits other NCS data flows to share the network. The utility of the network adaptive deadband involves not only IP network applications but also collaborative robotics in local networks, MMMS tele-operation or plants with several data flows.

The solution presented has a practical nature, but as a future line, a more theoretical depth is required to refine the deadband function and to analyze and model the network-dependent deadband. To do this, control system equations and transport models should be mixed. The research lines of event-triggering and the attempts to relate protocols and control design are interesting start points.

## Figures and Tables

**Table 1.** Deadband function for a Gantry Crane.

*ref_gc_*_1_ = 10 · *sin*(2*π*/15 · *t*) *ref_gc_*_2_ = 10 *· triang*(*T*= 18.1)		
	master to slave (ms)	slave to master (sm)
*th_n_*(*δ*)	$\frac{0.0035}{0.0035+\delta }$	$\frac{0.0035}{0.0035+\delta }$
(*δ_min_*, *δ_max_*)	(0, 0.25)	(0, 10)
*δ_c_*	0.02145	1
